# The present and future of neural interfaces

**DOI:** 10.3389/fnbot.2022.953968

**Published:** 2022-10-11

**Authors:** Davide Valeriani, Francesca Santoro, Marcello Ienca

**Affiliations:** ^1^Neurable Inc., Boston, MA, United States; ^2^Institute for Biological Information Processing - Bioelectronics, IBI-3, Forschungszentrum Juelich, Juelich, Germany; ^3^Faculty of Electrical Engineering and Information Technology, RWTH Aachen University, Aachen, Germany; ^4^College of Humanities, Swiss Federal Institute of Technology Lausanne (EPFL), Lausanne, Switzerland

**Keywords:** neural interface, neurotechnology, brain-computer interface, brain-machine interface, neuroethics, neuromorphic, policy, neuroscience

## Abstract

The 2020's decade will likely witness an unprecedented development and deployment of neurotechnologies for human rehabilitation, personalized use, and cognitive or other enhancement. New materials and algorithms are already enabling active brain monitoring and are allowing the development of biohybrid and neuromorphic systems that can adapt to the brain. Novel brain-computer interfaces (BCIs) have been proposed to tackle a variety of enhancement and therapeutic challenges, from improving decision-making to modulating mood disorders. While these BCIs have generally been developed in an open-loop modality to optimize their internal neural decoders, this decade will increasingly witness their validation in closed-loop systems that are able to continuously adapt to the user's mental states. Therefore, a proactive ethical approach is needed to ensure that these new technological developments go hand in hand with the development of a sound ethical framework. In this perspective article, we summarize recent developments in neural interfaces, ranging from neurohybrid synapses to closed-loop BCIs, and thereby identify the most promising macro-trends in BCI research, such as simulating vs. interfacing the brain, brain recording vs. brain stimulation, and hardware vs. software technology. Particular attention is devoted to central nervous system interfaces, especially those with application in healthcare and human enhancement. Finally, we critically assess the possible futures of neural interfacing and analyze the short- and long-term implications of such neurotechnologies.

## Macro-trends in neurotechnology development

Neurotechnology is an umbrella term used to refer to the broad spectrum of tools, systems, applications, and methods that can be used to read or influence brain structure, function, or activity in humans. While the utilization of one single term to denote this broad technological spectrum may provide semantic parsimony, identifying the main trends within neurotechnology development is crucial as different kinds of neurotechnologies may have different clinical applicability and thereby raise different ethical and policy questions. In this section, we identify three key conceptual distinctions, namely, interfacing vs. simulating the brain, neural recording vs. brain stimulation, and hardware vs. software in neurotechnology.

### Interfacing vs. simulating the brain

The design of neural interfaces first requires the identification of brain patterns that could be used to control an actuator, such as an arm prosthesis. This process can be approached in essentially two ways, namely, by directly measuring brain activity at different spatial and temporal resolutions, at rest or during the production of a task, or by simulating brain activity through mathematical modeling.

The classic approach is direct interfacing with the brain, which allows us to capture brain activity in real time. This approach has played a key role in advancing our understanding of brain function. This approach comes at a cost, as it requires (a) a technology to measure brain activity, which may have to be implanted, with associated costs and risks, and usually provides only a measurement scale; (b) a brain from which to measure it, human or animal, with all the associated ethical problems.

The increasing availability of neural data (Ienca et al., 2018a) combined with advances in mathematical modeling and machine learning is changing the way we investigate neuroscientific hypotheses. Simulation neuroscience is a new paradigm of brain research that aims at building a comprehensive digital model or copy of the brain (Fan and Markram, 2019). This approach has multiple advantages over traditional experimental neuroscience. Although it requires high computational power, it reduces overall experimental costs and alleviates ethical concerns associated with both animal and human experimentation compared with conventional research (Romeni et al., 2020). Moreover, it allows the simultaneous study of brain function at different scales (multi-scalar) and modes (multi-modal research). To date, researchers have been able to simulate the activity of some parts of the brain, such as the sensory cortex (Markram et al., 2015; Yamins and DiCarlo, 2016), the motor cortex (Pandarinath et al., 2018), the temporal cortex (Cadieu et al., 2014), and the visual cortex (Lindsay, 2020).

The next decade will likely be characterized by increased efforts in *system simulation neuroscience*, where different models will be integrated to progressively simulate the entire central nervous system. However, it is improbable to achieve such an ambitious goal by the end of the current decade. The integration process alone will require enormous effort to adapt individual models and will implicate in turn a wide range of other challenges to overcome (Makin, 2019).

### Neural recordings vs. brain stimulation

Just as humans combine the ability to read with the ability to write, brain-computer interfaces (BCIs) could also work by recording (“reading” in a metaphorical sense) brain activity or by stimulating (aka writing) the brain. Neural recordings could be obtained with a number of invasive or noninvasive methodologies, such as electroencephalography (EEG) and electrocorticography (ECoG). In all these circumstances, the BCI could only influence brain activity indirectly, that is, by modifying sensory stimuli, such as visual stimuli. Common BCIs based on neural recordings are spellers (Rezeika et al., 2018), as they generally use visual stimuli to elicit strong brain activity patterns and convert those brain signals into a letter to type on an interface. Conversely, brain stimulation allows the BCI to directly activate or inhibit certain brain areas *via* electrical stimulation. This leads to enhancements in human performance, for example, increasing vigilance (Nelson et al., 2014), reducing fatigue (McIntire et al., 2017), or increasing task performance (Nelson et al., 2015).

Most BCI systems employ only one interfacing modality: they either read from or write into the brain. However, in recent years, we have seen a few applications of bimodal BCIs, using multiple techniques to both record and stimulate the brain (McKendrick et al., 2015). These approaches will gradually become the new norm in the next few years, as brain stimulation combined with neural recordings enables next-generation applications of BCIs, such as direct communication between brains (O'Doherty et al., 2011).

### Software vs. hardware development

Advances in software technology were among the most significant developments in neural interfaces that we witnessed in the 2010's decade. New signal processing techniques, paired with a better understanding of both brain structure and function, allow to increase the signal-to-noise ratio of neural recordings and thereby to precisely measure brain activity against the noise. This progress may soon enable noninvasive BCIs to perform similarly to invasive ones, hence reducing health risks and financial costs for the patient. Moreover, advancements in machine learning and, particularly, deep learning have allowed neuroscientists to uncover novel brain features and build more complex classification models to handle highly dimensional input data (Craik et al., 2019; Roy et al., 2019).

Open-source software tools have also been instrumental in making these advances in signal processing and machine learning directly available to researchers and end-users. Among those, EEGLAB (Delorme and Makeig, 2004), OpenViBE (Renard et al., 2010), BCI2000 (Schalk et al., 2004), and MNE (Gramfort et al., 2014) are used daily in hundreds of research labs and neurotechnology companies around the world to speed up analysis and BCI prototyping.

Progress in hardware development has been much slower compared with software technology because of the high costs and time required for prototype development (Stieglitz et al., 2009). The Utah array was groundbreaking because it allowed the recording of large populations of neurons with a signal-to-noise ratio high enough to allow the development of BCIs for precise control (Maynard et al., 1997). Nevertheless, 25 years after its introduction, it is still the gold standard for this type of invasive brain recording. The majority of noninvasive BCIs still rely on EEG to record brain activity, a technology that was introduced about a century ago. That being said, one of the most promising developments in the brain recording area involves the gradual transition from wet to dry electrodes, which are cheaper and faster to set up and provide comparable measurements to wet sensors (Kam et al., 2019). Crucial progress has also been made in miniaturizing electronic components to build more powerful, efficient, and cheaper processing boards that provide the high computational power required for advanced neural interfaces.

Similar to open-source software, open hardware initiatives have also been instrumental in pushing innovation in electrical circuits for neural interfaces. In the BCI industry, such as OpenBCI and several BCI research labs (Rakhmatulin et al., 2021), have demonstrated how to build low-cost BCIs with consumer electrical components.

## Recent advances in neural interfacing

Among the various trends in neurotechnology, neural interfacing is of particular scientific, clinical, and ethical significance. Neural interfaces are devices that interact with the nervous system. In the following, we will articulate two main trends in neural interfacing, namely, neuroadaptive technologies and neurohybrid interfaces.

### Neuroadaptive technologies

**Figure 1:**
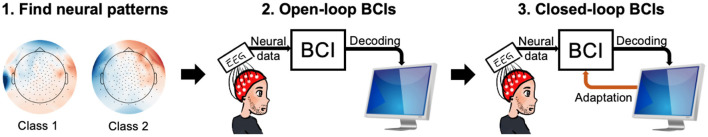
Stage of development of a BCI.

The development of BCIs usually involves three steps (Figure 1). First, the identification of strong patterns in neural activity that could be used to control the device. This stage involves the development of well-controlled laboratory experiments to ensure that patterns are present between subjects and experimental sessions. When such patterns are validated, open-loop BCIs are developed. These are systems in which similar patterns are tested in more realistic settings, but in which the user receives no feedback from the BCI (Shanechi, 2019). After optimizing the parameters of open-loop BCIs, the last step is to close the loop, providing the BCI with the ability to update its internal parameters in real time and adapt to the user's mental state. These neuroadaptive BCIs (Zander et al., 2016) are the most challenging to implement, but they are the ones that promise to provide the most seemingless user interaction.

The last decade has experienced exponential growth of open-loop BCIs in various domains, from traditional spellers for the disabled (Rezeika et al., 2018) to human enhancement (Cinel et al., 2019). For example, BCIs have been developed to decode our degree of confidence during decision-making and assist us to make better decisions in groups (Valeriani et al., 2017a).

Closed-loop BCIs have also been investigated, although to a lesser extent. For example, a BCI regulating arousal *via* auditory neurofeedback was developed in a flight simulator (Faller et al., 2019). Recent research has also enabled the possibility of developing closed-loop BCIs for therapeutic purposes, from controlling epileptic seizures (Maksimenko et al., 2017) to restoring lost emotional function in neuropsychiatric disorders (Shanechi, 2019).

### Neurohybrid interfaces

Closed-loop interfaces require optimal neuro-inspired functionalities (i.e., plasticity), as well as efficient power consumption and connectivity, similar to what happens in the neuronal tissue. In fact, the new generation BCIs and neuroelectronic platforms ultimately should resemble both the physical and electronic architectures and features of neuronal cells (Lubrano et al., 2020). While neuronal tissue engineering has greatly advanced in reconstituting biological neuronal networks from the single cell to scaffold-based tissue architectures, these platforms can be optimized *in vitro* and potentially be implanted *in vivo* to form functional connections over time.

On the contrary, electronic microdevices have been shown to be ideal platforms for both electrophysiology and stimulation to investigate and eventually restore lost electrical functionalities. Electrode-based solutions for deep brain stimulation have been demonstrated to achieve even profound areas of the brain and significantly affect the electrical activity of the neuronal tissue (Lozano et al., 2019). This is extremely relevant in neurodegenerative pathologies, such as Parkinson's disease, where implantable BCIs can successfully overcome major symptoms like tremor that massively affect a patient's daily life (Pulliam et al., 2020). However, these BCIs are mainly passive and lack local computing resources that can adequately contribute to a closed-loop (control).

In this scenario, neuromorphic platforms are emerging as the new frontier of BCIs as they resemble (neuro)electronic functions, such as short- and long-term potentiation and depression (Ham et al., 2021). Serving as key hardware for artificial neural networks, platforms like SpiNNaker, TrueNorth, and Loihi support closed-loop computing with low power consumption and miniaturized devices. For example, spiking neural networks have been used to continuously monitor brain activity and detect epileptic high-frequency oscillations with a low-power wearable device, paving the way to cheaper and less invasive epileptic monitoring (Burelo et al., 2022). Nevertheless, despite being low power, these neuromorphic platforms can do enough computations to accurately decode brain patterns typically associated with BCI paradigms, such as motor imagery (Behrenbeck et al., 2019).

In the attempt of creating optimal implantable BCIs for sensing and stimulating target brain areas, a major challenge is to pinpoint the tissue-device coupling to ensure a stable connection over time. This is strongly dependent on the engagement of the devices at the single-cell level, where the bidirectional communication and the physical interaction between the artificial and the biological counterparts are taking place.

Traditional platforms are based on metals and semiconductors that assemble in typical transistor-based devices required for operation with high electrical potentials, which differ in orders of magnitude from those of biological neurons. Moreover, it is highly demanding to extend the production of these devices to more conforming and flexible BCIs, which are required to physically couple the soft neuronal tissue (Jeong et al., 2020). In this scenario, organic neuromorphic platforms recently arose as tools to directly interface biological and artificial neurons to form functional biohybrid synaptic connections (Keene et al., 2020). Based on biocompatible organic semiconductors, these devices are capable of mixed ionic-electronic (trans)conduction that very accurately resembles the complexity of the neuronal electrochemical environment in which the neuronal bidirectional communication takes place (Burelo et al., 2022). Furthermore, their long-term potentiation and short-term depression (Tuchman et al., 2020) have been exploited to ultimately interface robotic actuators to comply with basic tasks in a closed-loop manner (Krauhausen et al., 2021).

In summary, neuromorphic platforms represent one of the most promising avenues of research to develop next-generation BCIs that seamlessly integrate with the human brain and require less power to operate.

## Proactive ethics for neural interfaces

Ethical reflections are inherent to neuroscience and neurotechnology since their very beginnings. This is due to the human brain being the fundamental site of life-maintaining functions (e.g., respiration), as well as mental faculties and processes, such as consciousness, memory, and perception. Therefore, the prospect of reading out from and/or writing into the brain raises the challenge of, respectively, revealing and influencing mental faculties and processes in a more direct manner compared with any other technology.

Since the 1990's, two fields of normative reflection on neuroscience and neurotechnology have arized, namely, neuroethics and neurolaw; the former focuses on ethical challenges, while the latter focuses on legal ones. Historically, the mainstream approach to ethical and legal assessments in neurotechnology has been reactive in character: reacting to the past (e.g., previously developed neurotechnological systems) and solving matters as they arise. The advantage of reactive approaches to the ethics of neurotechnology is that they allow ethicists and engineers to optimize their efforts and focus on concrete problems rather than on the anticipation of possible future scenarios that are often hard to foresee. However, reactive approaches–if pursued alone–present several disadvantages. First, they are structurally postdated since they provide ethical advice only at the post-development level, i.e., at a stage when there is less or no room for modification of a neurotechnology system. Second, in several domains of cognitive and physical disability, the lack of proactive ethical and social considerations has been inferred as a determinant of low adoption and acceptance of technology (Ienca et al., 2018c). In fact, if the impact of ethically relevant factors is not anticipated, products might not match the end-users' needs and wishes, hence resulting in sub-optimal uptake, implementation lag, and delayed clinical or social benefit. Third, there is a risk that lack of proactive ethical considerations may cause negative public perceptions or even unjustified anti-technological Luddite fears among end-users, caregivers, and other relevant stakeholders. This risk is particularly concrete in relation to advanced technologies, such as those that incorporate or embed artificial intelligence (AI), as their underlying mechanisms and functionalities are often unclear to users. Finally, reactive approaches are a possible source of antagonism and conflict between designers and developers, on the one hand, and ethicists and policymakers, on the other hand, as, in a reactive context, the work of the former professionals is being constantly questioned and judged by the latter.

In the light of the analysis presented above, we argue that a proactive ethical approach is best suited to anticipate the ethical challenges of neural interfaces and to ensure the ethical assessment of emerging neurotechnologies. In fact, in proactive assessment, ethical matters are addressed before they become an issue. This requires a foresight-oriented approach that focuses not only on short-term issues but also on long-term issues. Further, it requires an evidence-driven exploration of expected and alternative futures and guiding futures to inform strategy. Clarifying the time scale of proactive ethical assessments is of utmost importance as it is necessary to avoid policy confusion based on unrealistic expectations or conflation of the time scale of neurotechnology development.

## The next decade of neurotechnology

As we have seen earlier, in the past few decades, neuroscience has broadly focused on expanding our understanding and knowledge of the human brain. In parallel, engineering has focused on innovation in hardware and software to increase the amount of information we can gather from the brain. The next decade will likely be focused on the integration of these advancements in science and engineering to build novel neurotechnologies that will improve our lives. In this section, we discuss what we consider the most promising ones.

### Doctors 2.0

The combination of brain imaging with machine learning allows the development of decision-support systems that can help clinicians diagnose and treat neurological disorders. For example, *ad hoc* deep learning models can quickly and accurately diagnose a variety of brain disorders, including Parkinson's disease (Oh et al., 2020), Alzheimer's disease (Liu et al., 2014; Suk and Shen, 2015), epilepsy (Khan et al., 2021), dystonia (Valeriani and Simonyan, 2020), brain cancer (Tandel et al., 2019), and cerebral palsy (Zhu et al., 2021). Neurotechnologies will be more and more integrated in the clinics to help reduce the workload of clinicians and improve diagnostic accuracy.

In addition to augmenting diagnosis, neurotechnologies can also represent a novel treatment for brain disorders. For example, patients with epilepsy can currently be implanted with responsive neurostimulation technologies (NeuroPace, Inc.) that detect seizure onsets by continuously monitoring brain activity. When seizure-like activity is detected, the device automatically starts stimulating the brain by injecting small amounts of electrical current to stop or shorten the seizure. Several studies have shown that this technique reduces seizure occurrence by over 50% (Agostini et al., 2019; Krucoff et al., 2021). Neuromodulation represents the golden standard treatment for several other neurological disorders, including Parkinson's disease (Andrews, 2010). Integrated neurotechnologies capable of adjusting brain stimulation parameters in real time while monitoring brain activity represent the most promising technology for the clinical treatment of neurological disorders.

### Synthetic memory

Advancements in understanding how information is encoded in the brain allow the development of artificial decoders of individual memories (Rissman et al., 2010). These neurotechnologies can facilitate memory retrieval and improve how information is organized in the brain. For example, they can be used for helping eyewitnesses recall relevant memories before a trial, hence having a direct impact on policy (Vedder and Klaming, 2010).

Neurotechnologies can also help us overcome the limitations of our memory. For example, visual short-term memory has a limited item and information capacity (Sewell et al., 2014). The development of artificial memory leveraging the knowledge on encoding capabilities of the brain will allow us to restore (Berger et al., 2011) or even extend our memory capabilities (Garner et al., 2012; Vetere et al., 2019).

### Optimized communication

Brain-computer interfaces were invented to restore communication capabilities in people with severe disabilities (Wolpaw et al., 2002). For example, the P300 speller was designed to allow patients to type sentences on a computer screen using their brain activity, one character at a time (Farwell and Donchin, 1988).

This technology can then be paired with speech synthesizers to restore speech capabilities. Since their inception, BCIs for speech decoding have evolved at a fast pace, and current research suggests that we may be able to decode full sentences from minimally invasive brain recordings (Herff et al., 2019; Chang and Anumanchipalli, 2020; Makin et al., 2020; Angrick et al., 2021). This progress would not only make these devices broadly used as prosthesis for speech restoration for people with disabilities but also enable novel forms of communication that are more respectful of privacy, such as silent-speech interfaces (Denby et al., 2010).

### Neurally integrated prosthesis

A neurally controlled prosthesis is an artificial device replacing or enhancing a missing or impaired part of the body that is controlled by the nervous system of the user. Traditionally, these prostheses are controlled using electromyography (EMG) signals, which are picked up by electrodes placed over the peripheral muscle attached to the prosthesis. The rationale behind this approach is that EMG signals have a higher signal-to-noise ratio than signals captured from the brain and, so, are easier to process. However, an EMG-based prosthesis typically operates using residual muscles that do not convey full information about the movement to be performed. For example, a hand prosthesis often uses biceps and triceps EMG activity, which does not carry information about the opening or closing position of the hand (Parajuli et al., 2019).

The next generation of neurally controlled prosthesis will instead capture the detailed motor intent of the user from the brain activity (Nazarpour, 2020; Vilela and Hochberg, 2020). This will broaden the degrees of freedom of prostheses and boost control and integration with the human body. Current neural prostheses (Gilja et al., 2012, 2015) suffer from limited speed and control accuracy, which may be enhanced by the advances in software and hardware promised for the next decade, as well as more hybrid BCI approaches, which combine multimodal signals (e.g., EEG and EMG) or multiple BCI paradigms (e.g., P300 and SSVEP) to enhance the signal to noise ratio (Leeb et al., 2011; Li et al., 2013; Lin et al., 2016).

Advancements in neurally integrated prosthesis will also facilitate the use of BCIs for rehabilitation purposes. Several studies have shown the potential of BCIs in helping patients regain motor control after severe conditions, such as stroke (López-Larraz et al., 2018; Mane et al., 2020) or multiple sclerosis (Carrere et al., 2021). These effects are possible because BCIs can bypass the impaired neuromotor system and (re)train patients to gain control of the limbs (Robinson et al., 2021). The next decade will represent a unique opportunity to conduct large-scale clinical trials to prove the impact of neurally controlled prosthesis for effective motor rehabilitation.

### Augmenting intelligence and cognition

Another very promising area of application of neurotechnologies is cognitive augmentation (Cinel et al., 2019). This pertains to increasing human performance in higher-order brain functions, such as reasoning and decision-making. Often referred to as passive BCIs (Zander and Kothe, 2011), these neurotechnologies monitor brain activity and aid the user to gain insights into their cognitive function. For example, BCIs can be used to decode the decision made by a user (Luu and Chau, 2009; Tzovara et al., 2015) or to estimate how confident the user was in a decision (Poli et al., 2014), enabling groups to decide based on the most reliable members and boost their performance (Valeriani et al., 2017a,b).

The development of neurotechnologies to augment cognitive function will also accelerate the integration between humans and machines (Gao et al., 2021). While AI already masters tasks requiring heavy computations, such as the game of Go (Silver et al., 2017), humans still remain more accurate than AIs in tasks requiring reasoning and high-level computations. Yet, future neurotechnologies can help build human-AI teams that correct individual weaknesses and effectively augment human capabilities. For example, early results in face recognition suggest that these teams may perform more accurately than humans or AI alone (Valeriani and Poli, 2019).

### Putting all together: Everyday neurotechnology

Similar to general AI, i.e., artificial intelligence capable of performing multiple tasks and adapting to a changing environment, neurotechnology will also benefit from advancements in generalization techniques that would allow them to augment different human capabilities. These everyday neurotechnologies will be our new companions, helping us with our daily routine, from controlling external devices (e.g., light switches and smartphones) with our mind to monitoring our attentional level at work to increase productivity.

Everyday neurotechnology will add new constraints to neurotechnology development, including appearance, cost, setup procedure, risks, availability, and ethical considerations. This integration process among multiple neural technologies, as well as additional requirements, will likely extend development to over one decade. Nevertheless, the current effort of several industries and academic players in pushing this endeavor makes us believe we will still be able to see the first integrated prototypes of everyday neurotechnology by 2030.

## Preparing the ethical future of neurotechnology

A crucial problem in ethics and technology assessment is clearly specifying the foreseeable time frame of the technological capabilities that may generate ethical and societal concerns. As stated in the Collingridge Dilemma, ethics assessment and technology regulation efforts face a double-bind problem. On the one hand, the impacts of technology cannot be easily predicted until the technology is extensively developed and widely used. On the other hand, control or change is difficult when the impact can be reliably predicted because the technology has become entrenched. Therefore, the challenge of proactive ethics with regard to neurotechnology consists in providing evidence-based impact assessment before neurotechnology becomes entrenched, hence immune to regulatory control or change. Reliably specifying a foreseeable time frame has a twofold benefit. On the one hand, it can prevent the emergence of fear-mongering narratives related to potential neurotechnology-related harms that may not materialize for decades. On the other hand, it may prevent the postponement of much-needed ethical compliance and regulatory intervention whose implementation may be delayed due to the erroneous perception that concrete technology-induced harms are rather far-fetched.

To deliver this twofold benefit, we propose a foresight map that classifies ethical and societal risks based on the time frame in which they are expected to emerge and generate societal concern (see Table 1).

**Table 1:** Foresight map of neurotechnology-related ethical and societal risks.

**Issue**	**Description**	**References**
**Time frame: Present**		
Neurosecurity	Security vulnerabilities of neurodevices and neurotech-related datasets	Ienca and Haselager, 2016; Pugh et al., 2018; Rickli and Ienca, 2021
Algorithmic bias	Bias in AI algorithms embedded in neurodevices or used in analytics	Yuste et al., 2017; Schleidgen et al., 2022; Webb et al., 2022
Neurohype	Inflated and unrealistic marketing claims by neurotechnology companies, such as mental relaxation and cognitive enhancement	Purcell-Davis, 2013; Wexler and Reiner, 2019
Off-target effects of neurostimulation	Unintended collateral effects of neurostimulation.	Mantione et al., 2014; Bluhm and Cabrera, 2022
Suboptimal models of neurotechnology development	Lack of standards for user-centered and neurophenomenological considerations	Kögel et al., 2019; Meyer et al., 2021; Pfotenhauer et al., 2021
Dual-use of neurotechnology	Military research on neurotechnology and cooptation of civilian neurotechnology for non-peaceful aims	Tennison and Moreno, 2012; Ienca et al., 2018b
**Time frame: Short-term (by 2025)**		
Mental privacy violations in the broad sense	The drawing of privacy-sensitive inferences from brain data which is exacerbated by (a) the increasing prevalence of consumer neurotechnology devices, (b) the associated availability of brain-related datasets, (c) machine learning (especially DL) algorithms from predictive/retrospective analysis *via* reverse inference	Ienca et al., 2018a; Minielly et al., 2020; Ienca and Malgieri, 2022
Neurodiscrimination	Risk of discrimination based on neuroanatomical or neurofunctional traits revealed by neurotechnology	Ienca and Ignatiadis, 2020
**Time frame: Mid-term (by 2030)**		
Neurographic profiling	Discriminatory profiling of individual and groups based on neurological characteristics (analogous to psychographic profiling)	Schleidgen et al., 2022
Neurowarfare	Weaponization of neurotechnology for offensive warfare and systematic utilization of military neurotechnology in armed conflicts	Tennison and Moreno, 2012; Ienca et al., 2018b; Rickli and Ienca, 2021
Cognitive enhancement	Neurotechnologies used for extra-medical augmentation of cognitive functions raise challenges for fairness and equality	Roelfsema et al., 2018; Cinel et al., 2019
On-target effects of neurostimulation	Targeted modification of psychological and/or behavioral traits for non-medical reasons	Ienca and Andorno, 2017
**Time frame: Long-term (by 2040)**		
Mental privacy violations in the narrow sense	Unveiling of semantic or visual content of mental states *via* neurotechnology and brain-data analytics	Haynes, 2011; Shen, 2013; Ienca and Andorno, 2017

## Data availability statement

The original contributions presented in the study are included in the article/supplementary material, further inquiries can be directed to the corresponding author.

## Author contributions

All authors listed have made a substantial, direct, and intellectual contribution to the work and approved it for publication.

## Conflict of interest

Author DV is an employee of Neurable Inc. Author MI has served as an ethics advisor to the Council of Europe, the OECD, and Kernel. The remaining author declares that the research was conducted in the absence of any commercial or financial relationships that could be construed as a potential conflict of interest.

## Publisher's note

All claims expressed in this article are solely those of the authors and do not necessarily represent those of their affiliated organizations, or those of the publisher, the editors and the reviewers. Any product that may be evaluated in this article, or claim that may be made by its manufacturer, is not guaranteed or endorsed by the publisher.
